# Treatment With Methotrexate Associated With Lipid Core Nanoparticles Prevents Aortic Dilation in a Murine Model of Marfan Syndrome

**DOI:** 10.3389/fcvm.2022.893774

**Published:** 2022-06-10

**Authors:** Maria Carolina Guido, Natalia de Menezes Lopes, Camila Inagaki Albuquerque, Elaine Rufo Tavares, Leonardo Jensen, Priscila de Oliveira Carvalho, Thauany Martins Tavoni, Ricardo Ribeiro Dias, Lygia da Veiga Pereira, Francisco Rafael Martins Laurindo, Raul Cavalcante Maranhão

**Affiliations:** ^1^Laboratory of Metabolism and Lipids, Heart Institute (InCor) of the Medical School Hospital, University of São Paulo, São Paulo, Brazil; ^2^Laboratory of Hypertension, Heart Institute (InCor) of the Medical School Hospital, University of São Paulo, São Paulo, Brazil; ^3^Department of Cardiovascular Surgery, Heart Institute (InCor), Medical School Hospital, University of São Paulo, São Paulo, Brazil; ^4^Department of Genetics and Evolutionary Biology, Institute of Biosciences, University of São Paulo, São Paulo, Brazil; ^5^Laboratory of Vascular Biology, Heart Institute (InCor), Medical School Hospital, University of São Paulo, São Paulo, Brazil

**Keywords:** Marfan syndrome, lipid nanoparticle, inflammation, aortic dilation, adenosine

## Abstract

In Marfan syndrome (MFS), dilation, dissection, and rupture of the aorta occur. Inflammation can be involved in the pathogenicity of aortic defects and can thus be a therapeutic target for MFS. Previously, we showed that the formulation of methotrexate (MTX) associated with lipid nanoparticles (LDE) has potent anti-inflammatory effects without toxicity. To investigate whether LDEMTX treatment can prevent the development of aortic lesions in the MFS murine model. MgΔloxPneo MFS (*n* = 40) and wild-type (WT, *n* = 60) mice were allocated to 6 groups weekly injected with IP solutions of: (1) only LDE; (2) commercial MTX; (3) LDEMTX (dose = 1mg/kg) between 3rd and 6th months of life. After 12 weeks of treatments, animals were examined by echocardiography and euthanatized for morphometric and molecular studies. MFS mice treated with LDEMTX showed narrower lumens in the aortic arch, as well as in the ascending and descending aorta. LDEMTX reduced fibrosis and the number of dissections in MFS but not the number of elastic fiber disruptions. In MFS mice, LDEMTX treatment lowered protein expression of pro-inflammatory factors macrophages (CD68), T-lymphocytes (CD3), tumor necrosis factor-α (TNF-α), apoptotic factor cleaved-caspase 3, and type 1 collagen and lowered the protein expression of the transforming growth factor-β (TGF-β), extracellular signal-regulated kinases ½ (ERK1/2), and SMAD3. Protein expression of CD68 and CD3 had a positive correlation with an area of aortic lumen (*r*^2^ = 0.36; *p* < 0.001), suggesting the importance of inflammation in the causative mechanisms of aortic dilation. Enhanced adenosine availability by LDEMTX was suggested by higher aortic expression of an anti-adenosine A2a receptor (A2a) and lower adenosine deaminase expression. Commercial MTX had negligible effects. LDEMTX prevented the development of MFS-associated aortic defects and can thus be a candidate for testing in clinical studies.

## Introduction

Marfan syndrome (MFS) is an inherited disease of the connective tissue, with the autosomal dominant transmission, with an incidence of one case per 5,000 individuals (1, 2). Mutations in the fibrillin-1 gene account for more than 90% of MFS cases (3, 4). Fibrillin-1 is an extracellular matrix glycoprotein that is an essential component of microfibrils, the major constituent of the elastic fibers (5, 6). The fibrillin-1 molecule has a latent transforming binding protein (LTBP) domain that binds the transforming growth factor-β (TGF-β). Hence, the effects of TGF-β on the extracellular matrix are attenuated (7, 8). Fibrillin-1 mutations reduce the binding capacity of LTBP, resulting in increased TGF-β activity which contributes to the pathogenesis of MFS and other cardiovascular diseases (9–11).

The cardiovascular, skeletal, and ocular systems are predominantly involved in MFS clinical manifestations (12, 13). The progressive enlargement of the aorta, producing incompetence of the aortic valve as well as dissection and rupture of the vessel, is the leading cause of mortality in patients with MFS (14–16). The ascending aorta and aortic arch are the regions most affected by aneurysmal development due to their higher content of elastic fibers and the greater exposure to repetitive hemodynamic stress from the left ventricle (17, 18).

In patients with MFS in advanced stages, there is clear-cut involvement of an inflammatory process that contributes to the development of aortic aneurysms (19, 20). Nonetheless, to our knowledge, clinical trials on the treatment of patients with MFS with anti-inflammatory drugs have not been attempted, possibly due to the ineffectiveness of the current drugs or their toxicity, especially to the kidneys, elicited by prolonged drug use (21, 22).

Our laboratory has long been testing the use of lipid core nanoparticles (LDE) as drug carriers. LDE has a lipid composition similar to that of low-density lipoprotein (LDL) but is manufactured without the apolipoprotein (apo) B100 moiety of LDL. In contact with the plasma, LDE acquires apo E which, similar to apo B, is recognized by the LDL receptors (23–26). In neoplastic or inflamed tissues, upregulation of LDL receptors occurs, which allows internalization into cells of drugs carried in LDE at much higher amounts compared with the commercial presentation of these drugs (27). In several animal models, drugs carried in LDE showed increased therapeutic efficacy together with a drastic reduction in toxicity (28–32).

A formulation of the anti-inflammatory and anti-neoplastic agent methotrexate (MTX) associated with LDE (LDEMTX) was shown to be taken up by cultured cells 90-folds more intensively than conventional MTX (28). LDEMTX was successfully tested in several animal models of diseases with an important inflammatory component, such as atherosclerosis, rheumatoid arthritis, myocardial infarction, and heart transplantation (29–32). In those animal models, LDEMTX showed anti-inflammatory action much greater than conventional MTX. In addition, LDEMTX had marked antioxidant, antifibrotic, anti-apoptotic, and neoangiogenic actions, which were not attained by conventional MTX (29–32). In this case, the cell internalization of MTX, which occurs *via* folate receptors and determines the well-known poor cellular uptake of commercial MTX (33), was replaced by the LDL receptor-mediated endocytic pathway that internalizes LDEMTX. This profound change in the uptake mechanism improves the actions of MTX when associated with LDE. In the current study, it was hypothesized whether precocious treatment with LDEMTX could prevent the development of dilation, dissection, and other aortic lesions that develop in the MFS murine model.

## Methods

### Mice and Treatment Groups

An MFS mouse model, mgΔloxPneo from C129/sv background, was utilized in this study. These mice present a fibrillin-1 mutation, caused by the replacement of *Fbn1* gene exons 19–24 by a neomycin resistance expression cassette flanked by lox-P sequence (34). Mice were bred and maintained under controlled temperature and light conditions in a pathogen-free environment at the Central Animal Facility of the Medical School University of São Paulo and were fed the Nuvilab CR1 rodent chow (*N*uvital, Colombo, Brazil) and water ad libitum. In this study, 40 MFS and 60 wild-types (WT) male mice were used. Animals were randomly allocated to six groups: (1) WT-control (*n* = 25): treated with LDE only; (2) MFS-control (*n* = 12): treated with LDE only; (3) WT-MTX (*n* = 19): treated with 1 mg/kg/week of commercial MTX; (4) MFS-MTX (*n* = 13): treated with 1 mg/kg/week of commercial MTX; (5) WT-LDEMTX (*n* = 16): treated with 1 mg/kg/week MTX associated with LDE; and (6) MFS-LDEMTX (*n* = 15): treated with 1 mg/kg/week MTX associated with LDE. All treatments were administered intraperitoneally once a week from 3 months until completing 6 months of age (35).

After 12 weeks, echocardiography was performed and the animals were euthanized for both morphometric and protein expression analysis of the aorta (from the aortic root until to left subclavian artery).

All procedures were performed in accordance with the guidelines of the Brazilian College of Animal Research and conformed to the National Institutes of Health guidelines. The study protocol was approved by the Ethics Committee of the University of São Paulo Medical School Hospital (number 1002/2018).

### LDE Preparation and Association With MTX

Methotrexate (Fagron, Rotterdam, Netherlands) was derivatized to increase lipophilicity and improve the association of the drug with LDE. For derivatization, MTX was solubilized in dimethylsulfoxide and to this solution was added bromododecane and cesium carbonate. The mixture was stirred under an inert atmosphere for 24 h at room temperature (36). The reaction was monitored by ultrahigh-performance liquid chromatography (UHPLC) (*N*exera X2 Shimadzu, MO, USA) to analyze the conversion rate of the MTX. Immediately, water was added to the reaction, and the product was extracted with chloroform. Then, the organic phase was submitted to successive washes with saturated sodium chloride solution and dried with magnesium sulfate. The product was filtered and concentrated in a rotary evaporator (Rotavapor R-300, Buchi, Switzerland). Finally, the MTX derivative was precipitated in methanol, filtered, and dried in a vacuum desiccator.

As described previously (33), with the MTX derivative added to the mixture of lipids, LDE was prepared with lipid mixtures containing phosphatidylcholine (Lipoid, Ludwigshafen, Germany), esterified cholesterol (Aesar, MA, USA), non-esterified cholesterol (Fabrichem, Connecticut, USA), and triglycerides (Sasol Germany GmbH, Hamburg, Germany).

Emulsification of lipids with and without the drug was performed by adding 0.01 M Tris-HCl pH 8.05 buffer in a high-pressure homogenizer, Emulsiflex C5 (Avestin Inc., Ottawa, Canada). The particle size (50–70 nm) was measured by the dynamic light scattering method at 90 degrees angle using the ZetaSizer Nano ZS90 equipment (Malvern, Malvern, United Kingdom). The association rate of the drug to LDE was analyzed by ultra-high performance liquid-chromatography (UHPLC) (Nexera X2, Shimadzu, Duisburg, Germany) using the isocratic method on a C18 silica column, mobile phase methanol: acetonitrile (90:10 v/v), at 254 nm. The nanoparticles were sterilized by a polycarbonate membrane filter with a 0.22 μm pore diameter (Merck, Millipore, MA, USA) in laminar flow and stored at 4°C in sterile glass bottles.

### Echocardiography

The echocardiographic study was performed at the end of the protocol when the mice had completed 6 months of age. Mice were anesthetized with isoflurane 2%, and images were obtained using a Vevo 2100 system (Visual Sonics, Toronto, Canada) equipped with a 40 MHz probe. The echocardiographic measurements were performed according to the American Society of Echocardiography Guidelines. The bi-dimensional images of the ascending aorta, aortic arch, and descending aorta diameters were assessed by B-mode and measured from the suprasternal window in the longitudinal plane. All measurements were performed during three representative cardiac cycles of each animal. The analysis of echocardiography was performed blindly (15). Supplementary Figure 1 shows representative echocardiographic images of aortic diameter measurements of WT and MFS mice.

### Morphometry

After euthanasia, the aortas were sectioned immediately above the aortic valve, and the ascending aorta was formalin-fixed, paraffin-embedded, and cut into 5 μm sections. The area analyzed was the closest region to the aortic root. Tissue sections were stained with hematoxylin and eosin (HE), Picrosirius red, and Verhoeff's stain, and the morphometric studies were performed using an image analysis system (Leica Q500 IW; Leica Imaging Systems, Cambridge, UK).

The wall thickness of the aorta was assessed in HE stained sections. The lumen was quantified as the difference between areas delimited by external and internal elastic laminae (aortic lumen) under × 400 magnification.

Aortic dissection was measured by the percentage of the number of dissections per total number of aortic fragments obtained in tissue sections stained with Picrosirius red under 400 × magnification.

Aortic elastic fiber disruptions were quantified by counting the number of disruptions of elastic fibers per total aortic area in tissue sections stained with Verhoeff under 400 × magnification.

Aorta collagen volume fraction (CVF) was measured in Picrosirius red-stained sections as the percentage of red-stained connective tissue areas per total aortic area under 400 × magnification (35).

### Western Blot Analysis

The pool of ascending aorta, aortic arch, and descending aorta, (*n* = 4–5, per group) were homogenized in liquid nitrogen with RIPA lysis buffer and protease inhibitors (Thermo Fisher Scientific, MA, USA). The total protein of the samples was quantified in triplicate by the Bradford method in a multilabel plate reader (Victor X3, Perkin Elmer, CA, USA). About 50 μg of total protein was incubated in Laemmle sample buffer (Bio-Rad Laboratories, CA, USA). Proteins were size-fractionated on polyacrylamide/SDS gel. The polyacrylamide percentage was chosen according to the molecular weight of the targeted protein (Supplementary Table 1).

The separated proteins were transferred by electrophoresis to nitrocellulose membranes (Thermo Fisher Scientific, MA, USA). Membranes were blocked with 5% nonfat milk. Primary antibodies were incubated overnight and then the blots were washed and incubated with horseradish peroxidase-conjugated secondary antibodies (Calbiochem, CA, USA) (Supplementary Table 1). The detection was performed using an enhanced chemiluminescence method. The images were captured and analyzed by the Amersham Image 600 Imaging System (Amersham GE, CT, USA). The normalization of protein levels was performed using the β-actin expression, and the results were expressed as a percentage of the WT-control group means (35).

The cellular mechanisms involved in the aorta of MFS mice were studied by: (1) apoptosis: cleaved-caspase 3, Bcl-2 associated X protein (BAX), and B-cell lymphoma 2B-cell lymphoma 2 (Bcl-2); (2) inflammatory processes: T lymphocyte (CD3); macrophages (CD68), monocyte chemoattractant protein-1 (MCP-1), tumor necrosis factor-alpha (TNF-α), interleukin (IL)-1β, and IL-6; (3) metalloproteinase (MMP): 2 and 9; (4) fibrosis: collagen type-1; (5) angiogenesis: vascular endothelial growth factor (VEGF); (6) cell hypoxia: hypoxia-inducible factor-2α (HIF-2α); (7) TGF-β signaling pathway: pERK1/2/total ERK1/2 and pSMAD3/total SMAD3; and (8) adenosine signaling pathway: A1, A2a, A2b, and A3 receptors, kinase adenosine (ADK), and deaminase adenosine (ADA).

### Statistical Analysis

Data are expressed as means ± SEM. Data were analyzed using the test of normality by the Shapiro–Wilk test. For data that passed the normality test, we used one-way ANOVA complemented by Bonferroni's post-test. For data that did not pass normality, Kruskal–Wallis with Dunn's post-test was used. The mortality analysis was performed using the chi-square test, followed by Fisher's exact test. Linear regression was used to test the potential relationships between cellular mechanisms obtained by western blot analysis and the dilation of the ascending aorta, aortic arch, and descending aorta obtained by echocardiography analysis. Statistical analyses were carried out using GraphPad Prism v.8 statistical software (GraphPad Software Inc., CA, USA) and SPSS v.22 (IBM Software Inc., IL, USA).

## Results

### LDEMTX Restored the Weight Gain and Reduced Mortality in MFS Mice

Table 1 shows the bodyweight of the animals at the commencement of the study, when they were 3 months old; weight was equal in all six groups. The final weight and the weight gain were reduced in the MFS-control animal group as compared with WT-control (*p* < 0.05). LDEMTX treatment restored the weight gain in MFS-LDEMTX animals, whereas MTX treatment failed to achieve weight gain equal to WT-control (*p* < 0.05).

**Table 1:** Body weight, echocardiographic and morphometric parameters in WT and MFS mice with 6 months of age after MTX and LDEMTX treatments.

	**Control**		**MTX**		**LDEMTX**	
	**WT (*n* = 25)**	**MFS (*n* = 12)**	**WT (*n* = 19)**	**MFS (*n* = 13)**	**WT (*n* = 16)**	**MFS (*n* = 15)**
**Body weight**						
Initial (g)	24.4 ± 0.5	23.0 ± 0.5	23.9 ± 0.4	22.3 ± 0.9	23.3 ± 0.5	23.9 ± 0.4
Final (g)	27.1 ± 0.5	24.2 ± 0.7^a^	26.3 ± 0.5	23.1 ± 1.0^a^	25.6 ± 0.5	26.1 ± 0.4
Weight gain (%)	11.1 ± 0.1	5.2 ± 0.1^a^	10.0 ± 0.2	3.6 ± 0.4^a^	9.9 ± 0.5	9.2 ± 0.5
**Aortic dilation**						
Ascending aorta (μm/g)	58 ± 1	69 ± 2^b^	55 ± 1^c^	68 ± 2^b^,^d^	58 ± 2^c^	62 ± 2
Aortic arch (μm/g)	58 ± 1	82 ± 13^a^	56 ± 1^e^	71 ± 3^a^,^d^	59 ± 2^e^,^g^	64 ± 4^e^
Descending aorta (μm/g)	50 ± 1	60 ± 4^a^	48 ± 1^e^	64 ± 3^a^,^f^	52 ± 3^e^,^g^	56 ± 3
**Aortic remodeling**						
Wall thickness (μm^2^)	18.6 ± 1.3	19.3 ± 5.4	21.6 ± 1.6	24.2 ± 2.1	15.6 ± 2.5	20.4 ± 3.2
EFD (*n*umber/μm^2^)	0.4 ± 0.1	2.0 ± 0.3^a^	0.5 ± 0.2^e^	2.1 ± 0.2^a^,^f^	0.5 ± 0.1^e^,^g^	1.5 ± 0.2^a^,^d^,^h^
CVF (%)	2.7 ± 0.2	5.1 ± 0.3^a^	2.4 ± 0.6^e^	3.8 ± 0.4	2.2 ± 0.7^e^	3.5 ± 0.8
Aortic dissection (%)	0	34 ^a^	0 ^e^	28 ^a^,^f^	0 ^e^,^g^	12 ^a^,^e^,^f^,^g^,^h^

*WT, wild-type; MFS, marfan syndrome; MTX, methotrexate; EFD elastic fiber disruption; CVF, collagen volume fraction*;

a *p < 0.05 vs. WT-control*;

b *p < 0.01 vs. WT- control*;

c *p < 0.01 vs. MFS- control*;

d *p < 0.01 vs. WT-MTX*;

e *p < 0.05 vs. MFS- control*;

f *p < 0.05 vs. WT-MTX*;

g *p < 0.05 vs. MFS-MTX*;

h *p < 0.05 vs. WT-LDEMTX*.

Lipid nanoparticles methotrexate treatment reduced mortality in MFS mice (7%) compared with MFS-control (11%) and MFS-MTX (13%) groups [$X_{\left(1\right)}^{2}$ = 17.0; *p* < 0.01]. Most of those deaths were caused by aorta rupture at 10th and 11th weeks of age in MFS groups. There were no deaths in all three WT groups.

### LDEMTX Prevented Dilation and Dissection in the Aorta

**Figure 1:**
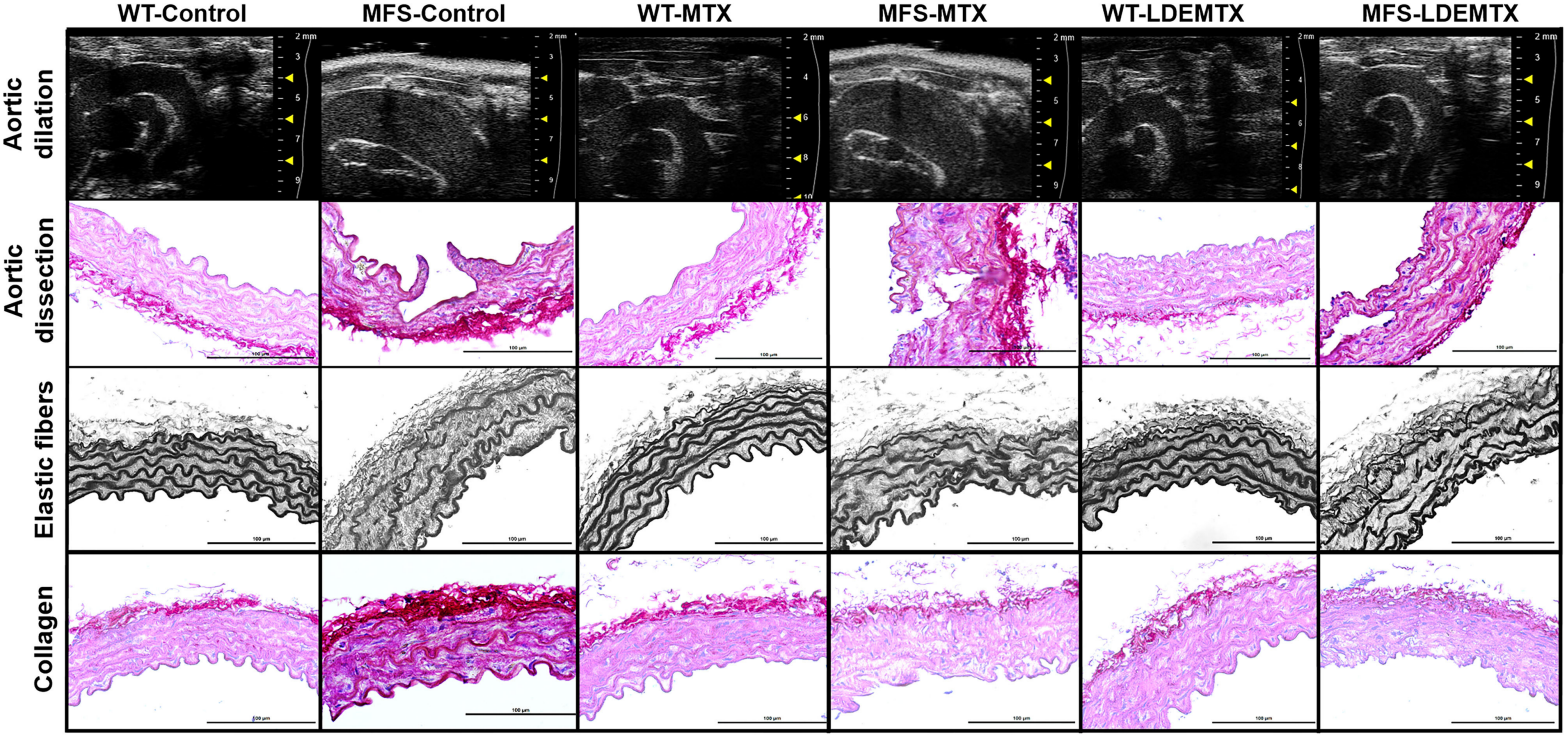
Representative echocardiographic and morphometric images in wild-type (WT) and Marfan syndrome (MFS) mice at 6 months of age, treated with methotrexate (MTX) and lipid nanoparticles methotrexate (LDEMTX), showing the dilatation and dissection, disorganization, and disruption of elastic fibers and fibrosis (in dark red) in the aorta.

The values and images of the echocardiographic exams performed at the end of the study when the animals were aged 6 months are shown in Table 1 and Figure 1. The MFS-control and MFS-MTX groups showed a greater diameter of the ascending and descending aorta and the aortic arch when compared with the three WT groups. On the other hand, LDEMTX treatment prevented the development of aorta dilation in all measured vessel segments.

Figure 1 shows representative images of aortic dissection obtained in tissue sections. The percentage of aortic dissections in animals treated with LDEMTX was roughly one-third of the MFS-control group, whereas MTX treatment was ineffective in reducing the appearance of aortic dissections (*p* < 0.05, Table 1).

### LDEMTX Reduced CVF in the Aorta

The structural remodeling represented by increased wall thickness, elastic fiber disruption, and CVF of WT and MFS animals was assessed in the aorta (Table 1 and Figure 1). There was no difference in aorta thickness between the WT and MFS groups and neither LDEMTX nor MTX treatments changed those parameters.

In the MFS-control group, there was a higher number of disruptions of the elastic fibers compared with the WT-control (*p* < 0.05), but the treatment with either LDEMTX or MTX did not change the occurrence of fiber disruptions in MFS (Table 1 and Figure 1). As seen in Figure 1, the content of collagen is increased in the aortas of MFS-control animals compared with those of WT-control. In Table 1, it is shown that treatment with both LDEMTX and MTX elicited a reduction of CVF (*p* < 0.05).

### LDEMTX Reduced Cleaved-Caspase 3 in the Aorta

**Figure 2:**
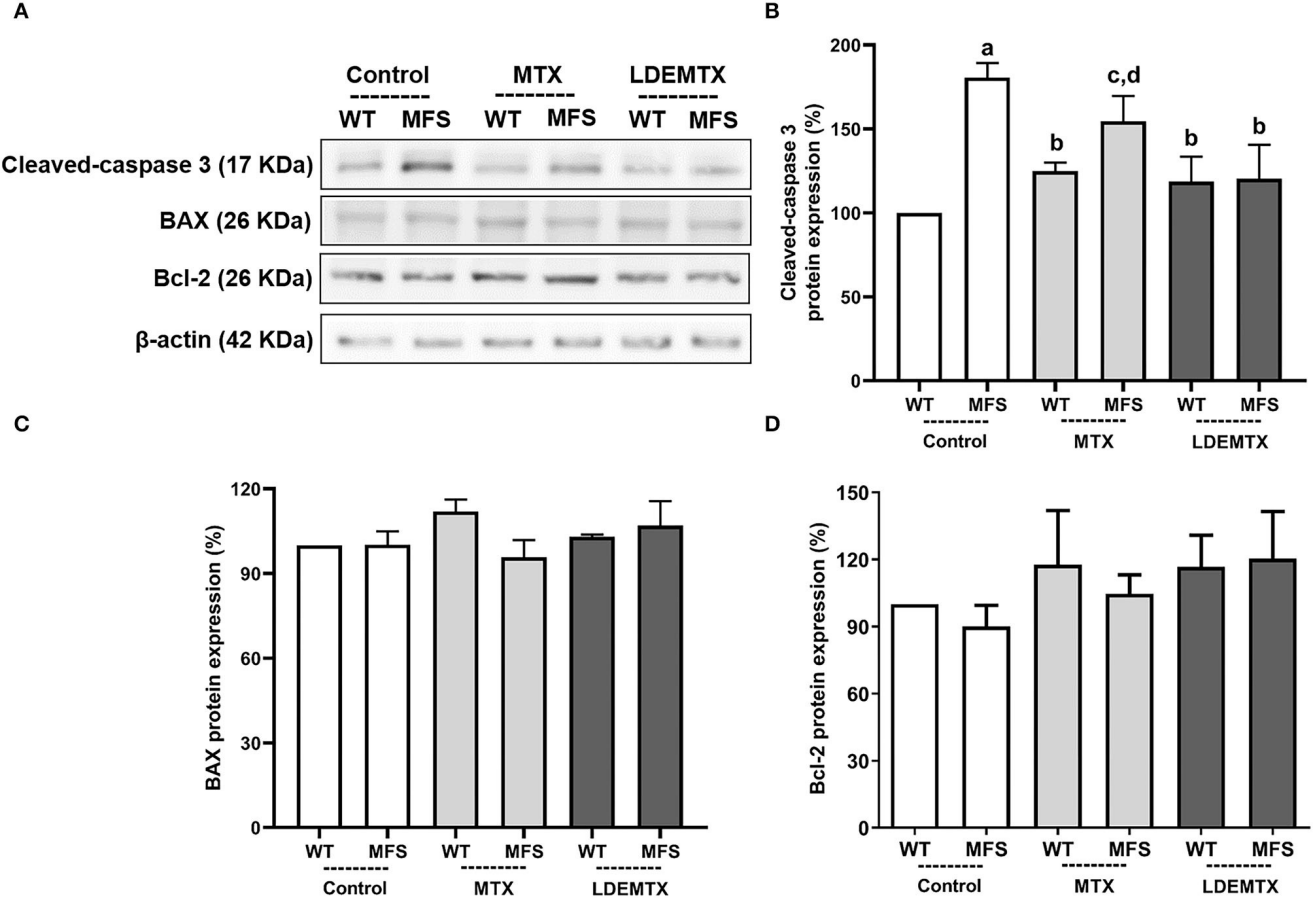
Representative bands **(A)** and graphs showing western blot analysis of pro-apoptosis cleaved-caspase 3 **(B)** and Bcl-2 associated X protein (BAX) **(C)** and anti-apoptosis Bcl-2 **(D)** protein expression in WT and MFS mice at 6 months of age, treated with MTX and LDEMTX. Values were normalized for expression of β-actin and results were expressed as a percentage of WT-control group means. Data are means ± SEM of a pool of aorta segments, comprising ascending aorta, aortic arch, and descending aorta (*n* = 4, per group). (a) *p* < 0.01 vs. WT-control; (b) *p* < 0.05 vs. MFS-control; (c) *p* < 0.05 vs. WT-control; and (d) *p* < 0.05 vs. WT-MTX.

Figure 2 shows the protein expression of pro-apoptosis cleaved-caspase 3 and BAX and anti-apoptosis Bcl-2 by western blot analysis in the aorta. Compared with WT-control, the expression of cleaved caspase 3 was increased (Figure 2B) in both MFS-control (*p* < 0.01) and MFS-MTX (*p* < 0.05), but not in the MFS-LDEMTX. Figures 2C,D show that protein expressions of BAX (*p* = 0.66) and Bcl-2 (*p* = 0.71) were not different among all the animal groups.

### LDEMTX Reduced Inflammation in the Aorta

**Figure 3:**
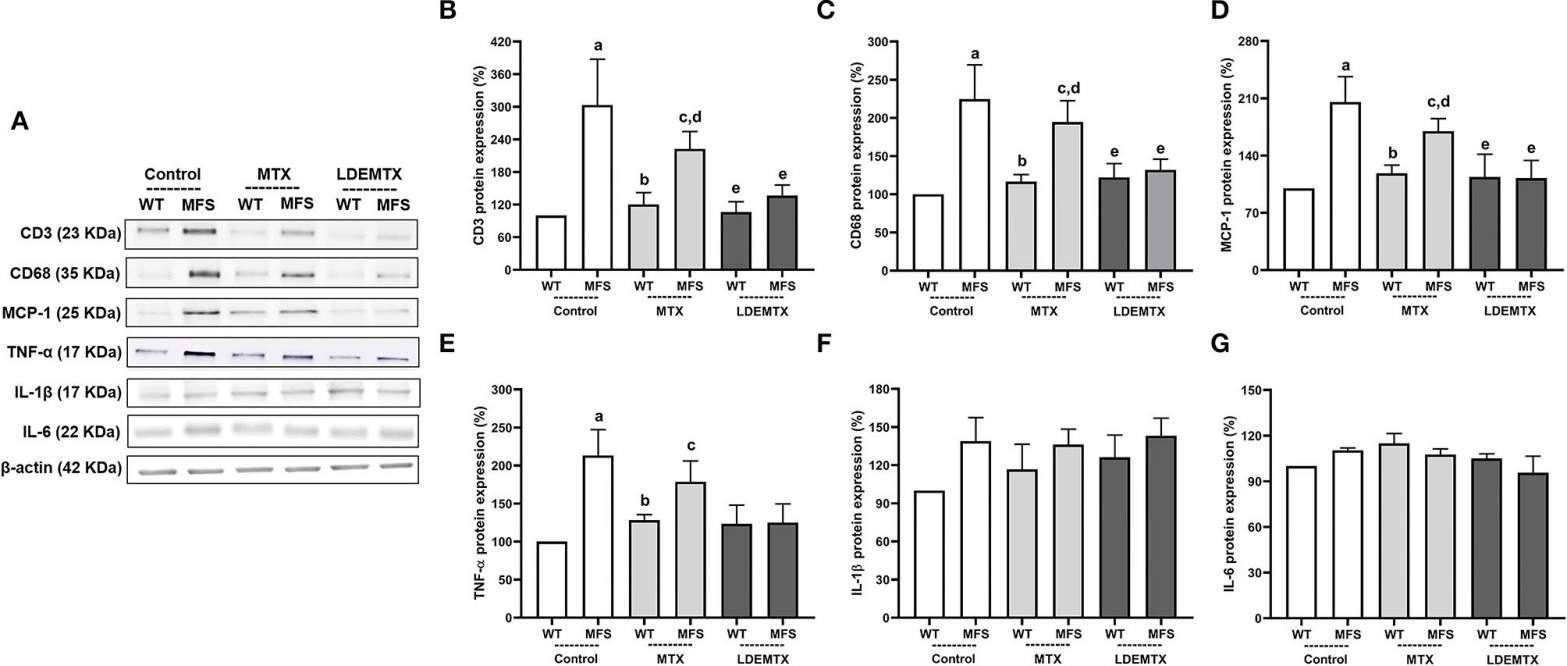
Representative bands **(A)** and graphs showing western blot analysis of a number of inflammatory cells T lymphocyte (CD3) **(B)** and macrophages (CD68) **(C)**, the chemokine monocyte chemoattractant protein*-*1 (MCP-1) **(D)**, and the cytokines proinflammatory tumor necrosis factor-α (TNF-α) **(E)**, interleukin (IL)-1β **(F)**, and IL-6 **(G)** protein expression in WT and MFS mice at 6 months of age, treated with MTX and LDEMTX. Values were normalized for the expression of β-actin and results were expressed as a percentage of WT WT-control group means. Data are means ± SEM of a pool of aorta segments, comprising ascending aorta, aortic arch, and descending aorta (*n* = 5, per group). (a) *p* < 0.01 vs. WT-control; (b) *p* < 0.01 vs. MFS-control; (c) *p* < 0.05 vs. WT-control; (d), *p* < 0.05 vs. WT-MTX; and (e) *p* < 0.05 vs. MFS-control.

Figure 3 shows the protein expression of CD3, CD68, and MCP-1, as well as pro-inflammatory cytokines TNF-α, IL-1β, and IL-6, quantified in the aorta by western blot analysis from micrographs of the three WT and the three MFS groups. Compared with the MFS-control group, LDEMTX treatment pronouncedly reduced the number of inflammatory cells, namely, lymphocytes and macrophages, as indicated by the decreased CD3 and CD68 protein expression (*p* < 0.05), so that the expression of those proteins was equal to that of the WT-control group (Figures 3B,C). Conventional MTX treatment had no effect.

The MCP-1 chemokine showed higher protein expression in MFS-control compared with WT-control (*p* < 0.01), which accounts for the increased number of inflammatory cells in the MFS animals. The LDEMTX treatment strongly reduced the MCP-1 expression in MFS (*p* < 0.05), whereas the MTX treatment had no effect (Figure 3D).

The expression of pro-inflammatory TNF-α was higher in the MFS-control animals than in WT-control (*p* < 0.01, Figure 3E). TNF-α expression in the MFS group was reduced by the treatment with LDEMTX, attaining values similar to those of WT. MTX treatment, however, did not reduce the values observed in the MFS group (*p* < 0.05, Figure 3E). The expressions of pro-inflammatory IL-1β (Figure 3F) and IL-6 (Figure 3G) did not differ between the MFS and WT control groups and were unchanged by either LDEMTX or MTX treatment.

### LDEMTX Reduced Collagen Type-1 in the Aorta

**Figure 4:**
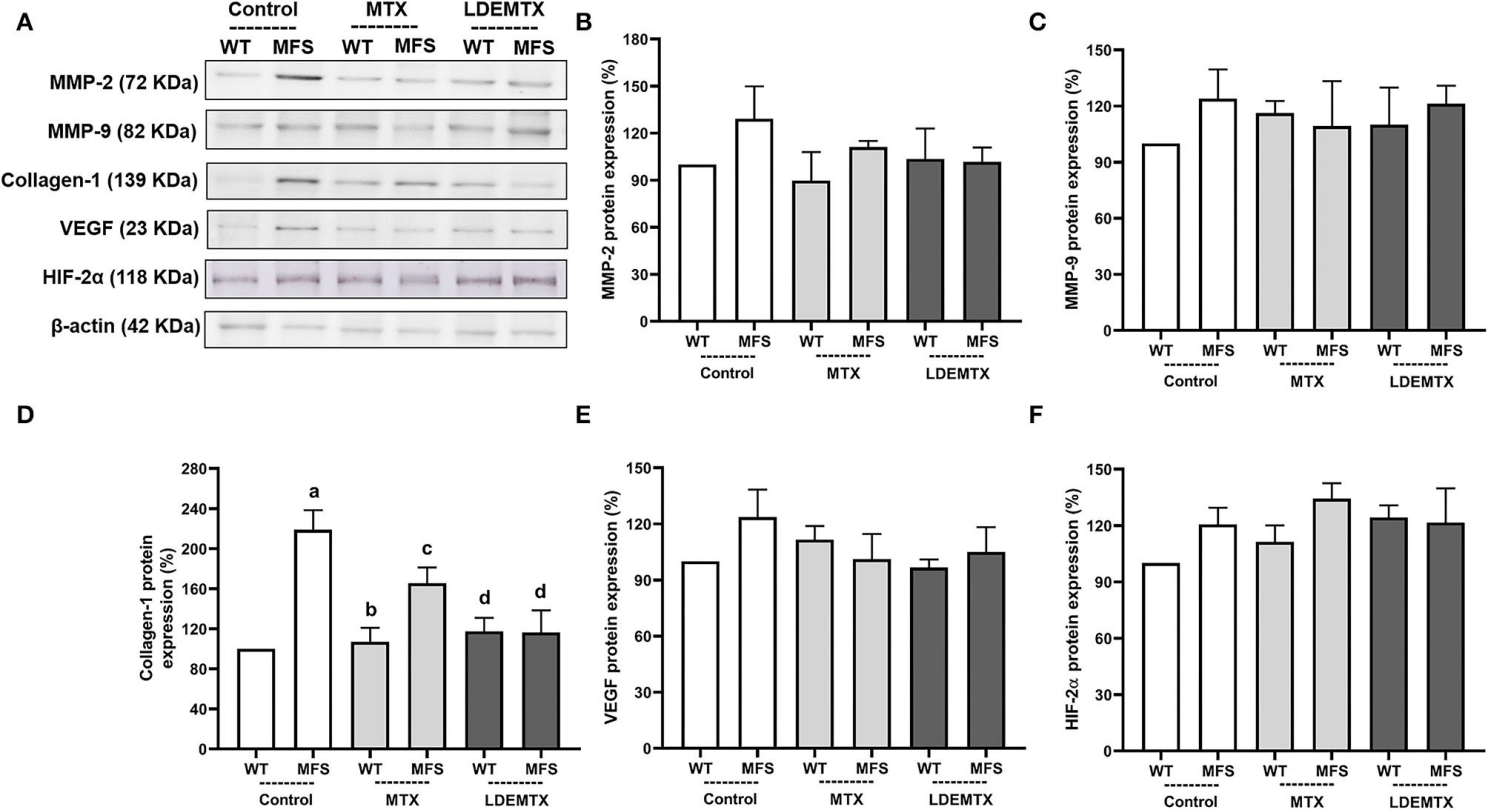
Representative bands **(A)** and graphs showing western blot analysis of metalloproteinase-2 (MMP-2) **(B)**, MMP-9 **(C)**, collagen type 1 **(D)**, vascular endothelium growth factor (VEGF) **(E)**, and hypoxia-inducible factor (HIF)-2α **(F)** protein expression in WT and MFS mice at 6 months of age, treated with MTX and LDEMTX. Values were normalized for the expression of β-actin, and results were expressed as a percentage of WT WT-control group means. Data are means ± SEM of a pool of aorta segments, comprising ascending aorta, aortic arch, and descending aorta (*n* = 4, per group). (a) *p* < 0.01 vs. WT-control; (b) *p* < 0.01 vs. MFS-control; and (c) *p* < 0.05 vs. WT-control.

The protein expression of MMP-2 and 9, collagen type 1, VEGF, and HIF-2α were analyzed in the WT and MFS treatment groups in the aorta by the western blot technique (Figure 4). There were no differences in MMP-2, MMP-9, VEGF, and HIF-2α protein expressions between WT and MFS groups (Figures 4B,C,E,F).

The protein expression of collagen type-1 was increased in MFS-control (*p* < 0.01) compared with the WT-control group. The treatment with LDEMTX reduced the expression of collagen type-1 in the aorta of MFS (*p* < 0.05). MTX treatment, however, did not change collagen type-1 expression, as shown by comparing the values of MFS-MTX with those of the MFS-control group (Figure 4D).

### LDEMTX Reduced TGF-β Signaling Pathway in the Aorta

**Figure 5:**
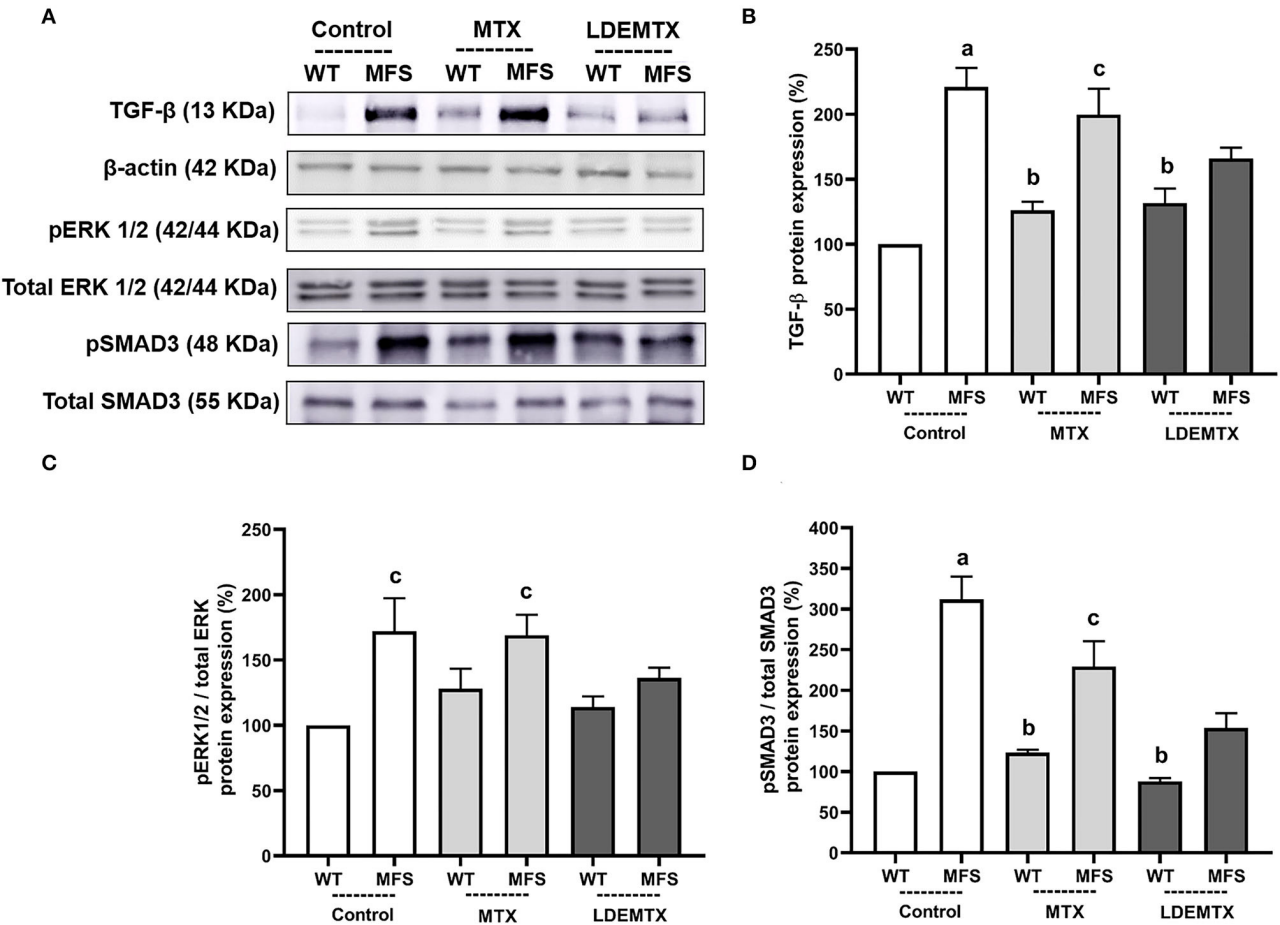
Representative bands **(A)** and graphs showing western blot analysis of transforming growth factor (TGF)-β **(B)**, phosphorylated extracellular signal-regulated kinases ½ (pERK1/2)/total ERK1/2 **(C)**, and pSMAD3/total SMAD3 **(D)** protein expression in WT and MFS mice at 6 months of age, treated with MTX and LDEMTX. Values were normalized for the expression of β-actin, and results were expressed as a percentage of WT WT-control group means. Data are means ± SEM of a pool of aorta segments, comprising ascending aorta, aortic arch, and descending aorta (*n* = 4, per group). (a) *p* < 0.01 vs. WT-control; (b) *p* < 0.01 vs. MFS-control; and (c) *p* < 0.05 vs. WT-control.

Figure 5 shows the protein expression of TGF-β, pERK1/2/total ERK1/2, and pSMAD3/total SMAD3, as quantified from micrographs of the aorta of WT and MFS mice groups by western blot analysis.

Transforming growth factor-β, pERK1/2/total ERK1/2, and pSMAD3/total SMAD3 protein expressions were higher in MFS-control than in the WT-control group. Treatment of MFS with LDEMTX diminished the expression of TGF-β, pERK1/2/total ERK1/2, and pSMAD3/total SMAD3 so there was no difference in those values between MFS-LDEMTX and the WT-control group. MTX treatment did not affect the expression of all those proteins (Figures 5B–D).

### LDEMTX Increased A2a Adenosine Receptor and Decreased ADA in the Aorta

Methotrexate pharmacological actions are related to the MTX's ability to release adenosine. In WT and MFS control groups, the release of adenosine was indirectly estimated by the protein expression of A1, A2a, A2b, and A3 adenosine receptors and by the protein expression of anti-adenosine kinase (ADK) and anti-adenosine deaminase (ADA), which are adenosine catabolic enzymes that catalyze the rapid phosphorylation of adenosine to 5′-monophosphate (AMP) and of inosine.

**Figure 6:**
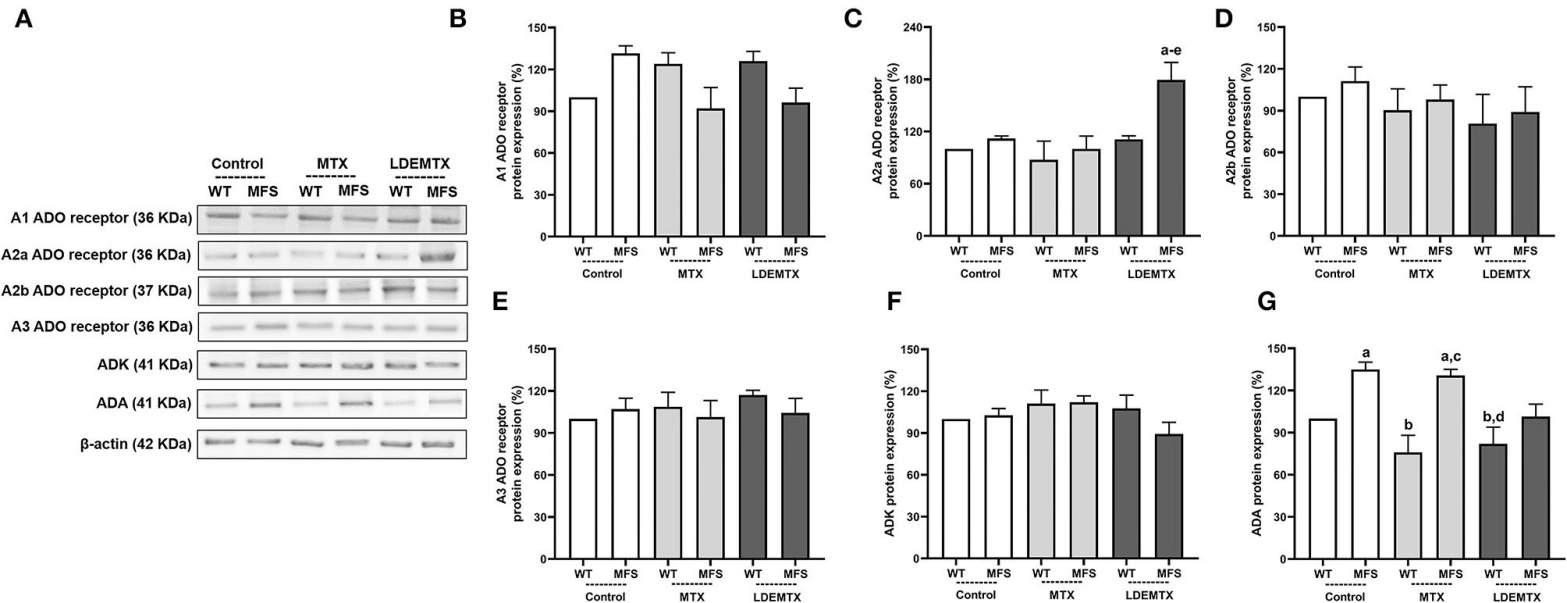
Representative bands **(A)** and graphs showing western blot analysis of anti-adenosine A1 receptor (A1) **(B)**, anti-adenosine A2a receptor (A2a) **(C)**, anti-adenosine A2b receptor (A2b) **(D)**, and anti-adenosine A3 receptor (A3) **(E)**, adenosine receptors and by anti-adenosine kinase (ADK) **(F)**, and anti-adenosine deaminase (ADA) **(G)** protein expression in WT and MFS mice at 6 months of age, treated with MTX and LDEMTX. Values were normalized for the expression of β-actin, and results were expressed as a percentage of WT WT-control group means. Data are means ± SEM of a pool of aorta segments, comprising ascending aorta, aortic arch, and descending aorta (*n* = 5, per group). (a) *p* < 0.05 vs. WT-control; (b) *p* < 0.05 vs. MFS-control; (c) *p* < 0.05 vs. WT-MTX; (d) *p* < 0.05 vs. MFS-MTX; and (e) *p* < 0.05 vs. WT-LDEMTX.

There was no difference in A1, A2b, A3 receptors, and ADK protein expression between aortas of WT and MFS control groups (Figures 6B,D–F). On the other hand, ADA expression was higher in the MFS-control group than in the WT-control group (*p* < 0.05, Figure 6G).

Treatment with LDEMTX increased the protein expression of A2a adenosine receptor in MFS mice (*p* < 0.05, Figure 6C) but did not affect A1, A2b, A3 receptors, and on ADK proteins. Furthermore, LDEMTX treatment modulated ADA protein expression down to normal values when compared with the WT-control group (*p* < 0.05, Figure 6G). These data indirectly suggest that the availability of adenosine in the aorta of MFS was increased by the LDEMTX treatment.

With respect to the MTX treatment, no changes in the expression of A1, A2a, A2b, A3 receptors, ADA, and ADK were observed (Figures 6B–F).

### Inflammatory Cells Are Involved in the Dilation of the Aorta

**Figure 7:**
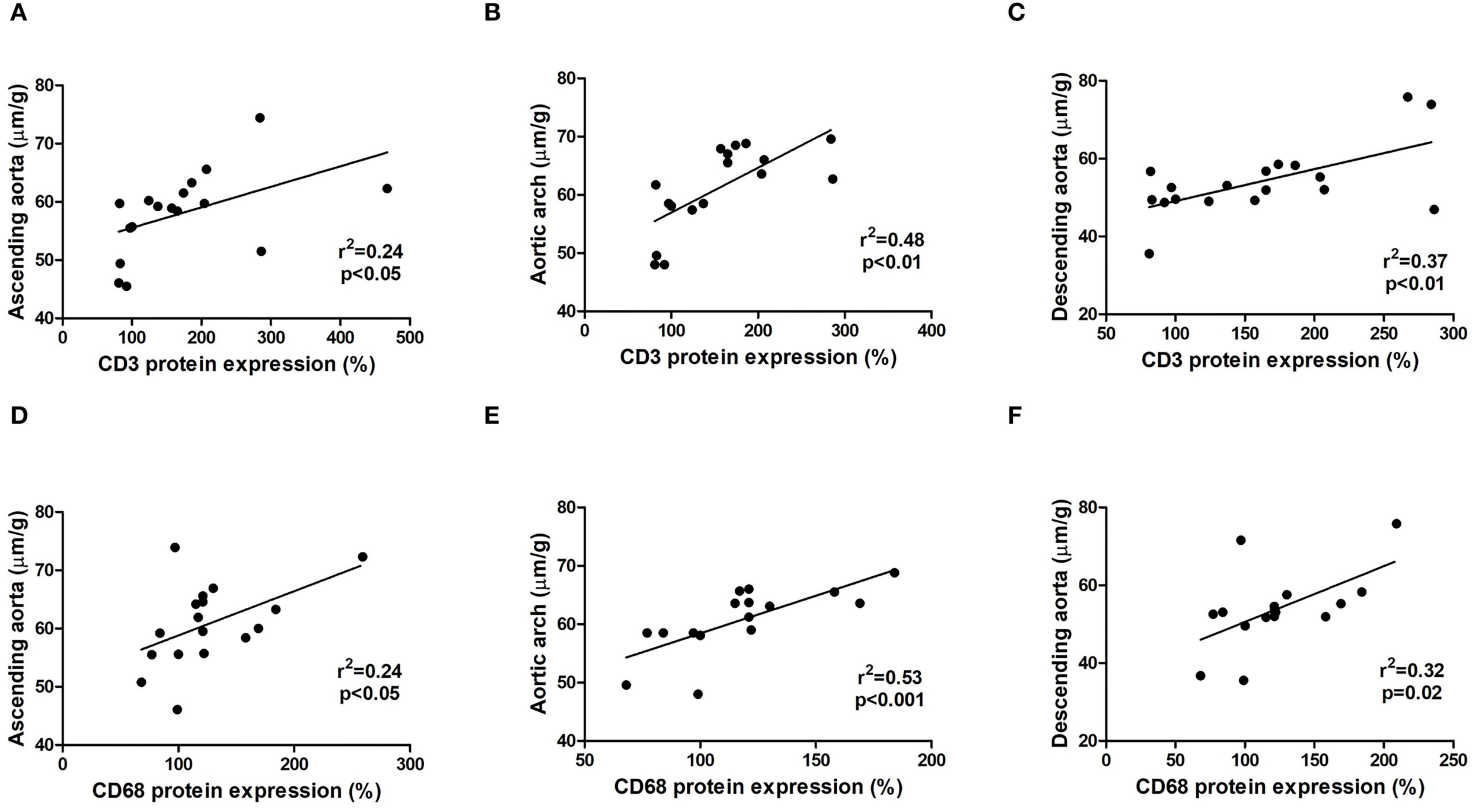
Relationship between inflammatory cells CD3 **(A–C)** and CD68 **(D–F)** protein expression obtained by western blot and the dilation of the ascending aorta, aortic arch, and descending aorta obtained by echocardiography analysis in WT and MFS mice at 6 months of age, treated with LDE, MTX, and LDEMTX.

To assess possible cellular mechanisms involved in the dilation of the aorta that occurred in the MFS group, linear correlation analysis was carried out with the data of vessel diameter of the ascending aorta, aortic arch, and descending aorta obtained by echocardiography vs. protein expression of cleaved-caspase 3, CD3, CD68, MCP-1, TNF-α, and collagen type-1. In this analysis, we solely found positive correlations between CD3 (Figures 7A–C) and CD68 (Figures 7D–F) expressions with the diameter of the ascending aorta, aortic arch, and descending aorta.

## Discussion

In this study, treatment with LDEMTX succeeded in preventing the appearance of the aortic dilation and dissection that are the hallmarks of MFS disease. Among the five murine models of MFS described in the literature, the current model has the closest resemblance to human MFS. Heterozygous mgΔloxPneo mice present the skeletal, pulmonary, and vascular abnormalities and recapitulate the clinical heterogeneity of human MFS (34, 35, 37). In this murine model, the aortic dilation and dissection usually start to appear at 3 months of age, and henceforth an aneurysmal rupture of the aortic arch occurs, which accounts for the high mortality rate of the animals between their 8th and 10th months of age (35). In this setting, the commencement of the treatment in the 3rd month of age resulted in an adequate timing for successfully preventing, in this experimental model, the occurrence of the most fatal disorder associated with MFS. The effectiveness of the LDEMTX in preventing the harms of MFS conceivably resulted in greater weight gain and a marked reduction of the mortality rates in the group of treated animals.

The results of the inflammatory parameters measured in this study fully confirm the potent effect of LDEMTX in reducing the chronic inflammation associated with MFS. Different inflammatory pathways were targeted by the treatment since not only the presence of macrophages but also of T lymphocytes was inhibited as also the secretion of MCP-1 and TNF-α. Together with these findings, the positive correlation found here between the expression of proteins of the inflammatory cells and the aortic diameters strongly suggests that the prevention of the aortic defects in MFS mice by the LDEMTX treatment is accounted for by the anti-inflammatory actions of LDEMTX.

Current treatments for MFS consist of the use of β-blockers and inhibitors of angiotensin-converting enzyme or angiotensin II receptors and surgical interventions to correct aortic dilations (38–41). Altogether, these therapies have considerably extended the life expectancy of patients with MFS which was formerly approximately 40 years. Some authors have tested the use of anti-inflammatory drugs in MFS murine models (42, 43). Treatments with methylprednisolone and with abatacept, a T-cell-specific inhibitor, were ineffective in decreasing aortic defects, such as wall thickness or elastic lamina breaks in C1039G Marfan mice (42). On the other hand, indomethacin prevented the appearance of aortic abnormalities in mgR MFS mice (43). The authors credited this effect for the ability of indomethacin to block the elastin degeneration, decrease the infiltration of macrophages, and the expression of MMP, as well as to the reduction of the activity in the aortic wall of both TGF-β and COX-2.

In our study, the protective effects of LDEMTX against MFS abnormalities can also be related to the observed inhibitory action of this formulation on pro-inflammatory cytokines. The several cytokines evaluated here are associated with monocyte recruitment and monocyte-macrophages activation, endothelial dysfunction, increase in migration of smooth muscle cell (SMC), reactive oxygen species (ROS) formation, MMP activity, and contribute to the formation of the aortic aneurysm (19, 20). In fact, in murine models of MFS, the protein expression of TNF-α, IL-6, and MCP-1 were reportedly involved in the development of the early phase of aortic dilation. In the phase of aneurysmal healing, IL-6 and MCP-1 can have an important role as mediators of the wound healing process (44–47).

In the C1039G murine model, caspase inhibition attenuates the development of aneurysms. Caspases are expressed on the cell surface of SMCs and have a role in the breakdown of elastic fibers, leading to extracellular matrix degradation. Our finding that LDEMTX treatment reduces cleaved-caspase 3 expression in the MFS mouse model mgΔloxPneo corroborates the findings of those authors (48, 49). The status of BAX and Bcl-2, which have pro and anti-apoptosis effects, respectively, has not been described in previous studies on MFS murine models. Noteworthy was the fact that the expression of those proteins was not altered in MFS control animals and that the LDEMTX treatment was without effect.

The treatment with LDEMTX of MFS animals reduced fibrosis as expressed by the reduction of collagen volume fraction and the protein expression of type I collagen of the aorta. This is a crucial finding to explain the general improvement of the aortic disease effects in the MFS animals. Collagen provides support and elasticity to the extracellular matrix of the vessel, and collagen overexpression is indicative of the presence of fibrotic disease. TGF-β is pro-fibrogenic cytokine, and excessive release of this cytokine induces collagen I production and contributes to aortic stiffness, affecting the mechano-transduction properties of the vascular cells and thus contributing to dilation of the ascending aorta (34, 37, 50–52).

Fragmentation of the elastic fibers is considered the main event leading to aortic dilation, and activation of MMPs is associated with this deleterious process in the MFS controls (53–55). Nonetheless, differences in the MMP-2 and MMP-9 protein expression were not observed among the six animal groups. However, it is worthwhile to mention that in the MFS mice model, most aortic aneurysms evolve mechano-signaling deregulation. This results in an impaired capacity of force generation of the vascular SMCs. Altogether, those mechanisms contribute to aortic dilation and dissection independent of the MMPs expression or elastic fiber breakage (56).

In MFS and other patients with aortic aneurysms, the proteomic array and Western blot analysis of aneurysm fragments did not differ from healthy aorta fragments in terms of VEGF expression, similar to our findings in the mgΔloxPneo murine model (57, 58). Treatment of the MFS mice with LDEMTX had no effect on both VEGF expression and HIF-2α, the cell hypoxia marker. Interestingly, in rats with acute myocardial infarction, the LDEMTX treatment induced an increase in VEGF and a decrease in HIF-2α protein expression, resulting in a reduction of the infarcted area (31).

An indirect way to estimate the availability of adenosine consists of the quantification of the adenosine receptors and the related catabolic enzymes (59). In the MFS controls, the expression of A1, A2b, and A3 adenosine receptors and of the ADK catabolic enzyme was not different from that of the WT mice. Nonetheless, the LDEMTX group showed increased A2a adenosine receptor and decreased ADA protein expression, compared with the MFS controls, suggesting that the treatment decreased the inflammatory process by increasing the availability of adenosine. MTX inhibits the activity of 5-aminoimidazole-4-carboxamide ribonucleotide (AICAR) transformylase, promoting the release of adenosine by a variety of cell types and tissues, which endows the anti-inflammatory action of this drug (60, 61).

The correlation found in our study between the protein markers of macrophages and T lymphocytes (CD68 and CD3, respectively), on one hand, and the diameter of the aorta, on the other hand, confirms the importance of the inflammatory process in the genesis of the aortic dilation characteristic of MFS. Taken together, our results indicate that the capability of LDEMTX to inhibit the appearance of aortic dilations can be ascribed to the potent anti-inflammatory action of this formulation, mediated through adenosine release. Noteworthy was the fact that the correlation between inflammatory cell markers and aortic diameter prevailed in all three segments of the aorta, namely, ascending, descending, and the aortic arch.

In contrast with LDEMTX, the small impact of the treatment with commercial MTX on the several anatomical, biochemical, and histological features was observed in the mgΔloxPneo murine model. Reduction in the collagen volume fraction was the sole statistically significant action of MTX found here. This remarkable difference in the pharmacological action of the two different formulations of this drug can be ascribed to the several-fold increase in the cell uptake of MTX when the drug is associated with LDE.

The current results highlight the nanomedicine approach to therapeutics: existing drugs can have their pharmacological properties and toxicity levels drastically changed by association with nanoparticles. It is noteworthy that while LDEMTX achieved marked pharmacological responses in the mgΔloxPneo murine model, the effects of commercial MTX were rather scanty, restricted to CVF reduction. Association with LDE determines numerous and marked changes in MTX biodistribution, pharmacokinetics, cell uptake, and concentration of the drug in inflamed target tissues. In this setting, to the end of attaining the beneficial effects observed here with LDEMTX, it would perhaps be necessary for the administration of much higher doses of commercial MTX, which mostly would compromise tolerability to the animals.

As a limitation of the study, the pronounced reduction of the toxicity of LDEMTX, which was documented in other animal models, should be studied in the mgΔloxPneo mice. LDEMTX treatment of animals with fully developed aneurysms should also be approached in future studies, since our protocol exclusively addresses the preventive actions of this treatment.

In conclusion, this study shows that treatment with LDEMTX prevented the development of aortic dilation and dissection in MFS mice. This therapeutic action was the result of LDEMTX in reducing inflammation, apoptosis, and fibrosis in the aorta, conceivably by increasing the availability of intracellular adenosine. Our data suggest that LDEMTX may be a candidate for clinical tests for the treatment of patients with MFS, aiming to further increase their life expectancy and quality of life.

## Data Availability Statement

The raw data supporting the conclusions of this article will be made available by the authors, without undue reservation.

## Ethics Statement

The study protocol was approved by the Ethics Committee of the University of São Paulo Medical School Hospital (Number 1002/2018).

## Author Contributions

MG: conceptualization, methodology, validation, formal analysis, resources, investigation, data curation, and writing—original draft supervision. NL: methodology, validation, investigation, data curation, formal analysis, and writing—original draft. CA: methodology, validation, investigation, and data curation. ET: investigation and data curation. LJ: methodology, validation, and investigation. PC: methodology, investigation, resources, and writing—reviewing and editing. TT: methodology and investigation. RD: writing—original draft. LP: resources and writing—original draft. FL: conceptualization, formal analysis, investigation, writing—original draft, and supervision. RM: conceptualization, investigation, writing—original draft, supervision project administration, and funding acquisition. All authors contributed to the article and approved the submitted version.

## Funding

This work was supported by the São Paulo Research Foundation (FAPESP, Grant 2014/03742-0, Brazil), the National Council for Scientific and Technological Development (CNPq, Grant 431290/2016-4, Brasilia, Brazil), and the National Institute of Science and Technology Complex Fluids (INCT-FCx, Brazil). Dr. Maranhão has an A-1 Research Carrier Award from CNPq.

## Conflict of Interest

The authors declare that the research was conducted in the absence of any commercial or financial relationships that could be construed as a potential conflict of interest.

## Publisher's Note

All claims expressed in this article are solely those of the authors and do not necessarily represent those of their affiliated organizations, or those of the publisher, the editors and the reviewers. Any product that may be evaluated in this article, or claim that may be made by its manufacturer, is not guaranteed or endorsed by the publisher.

## Supplementary Material

Supplementary Figure 1 The bi-dimensional images of **(A)** wild-type and **(B)** Marfan syndrome mice indicating the ascending aorta (1), aortic arch (2), and descending aorta (3) diameters were assessed by B-mode and measured from the suprasternal window in the longitudinal plane.

Supplementary Table 1 Western blot methods used in this study.

## Abbreviations

A1: anti-adenosine A1 receptor
A2a: anti-adenosine A2a receptor
A2b: anti-adenosine A2b receptor
A3: anti-adenosine A3 receptor
ADA: anti-adenosine deaminase
ADK: anti-adenosine kinase
ADO: adenosine
Apo: apolipoprotein
BAX: Bcl-2 associated X protein
Bcl-2: B-cell lymphoma 2
CD3: T lymphocyte
CD68: macrophages
EFD: elastic fiber disruption
ERK1/2: extracellular signal-regulated kinases ½
HE: hematoxylin and eosin
HIF: hypoxia-inducible factor
IL: interleukin
LDE: lipid nanoparticles
LDL: low density lipoprotein
LTBP: latent transforming binding protein
MCP-1: monocyte chemoattractant protein*-*1
MFS: Marfan syndrome
MMP: metalloproteinase
MTX: methotrexate
TGF: transforming growth factor
TNF-α: tumor necrosis factor-α
UHPLC: ultrahigh-performance liquid chromatography
VEGF: vascular endothelium growth factor
WT: wild-type.
