# Supplementary figures and images for: Treatment With Methotrexate Associated With Lipid Core Nanoparticles Prevents Aortic Dilation in a Murine Model of Marfan Syndrome
Source: Front Cardiovasc Med. 2022 Jun 10;9:893774. doi: 10.3389/fcvm.2022.893774 (PMC9226570; doi:10.3389/fcvm.2022.893774)

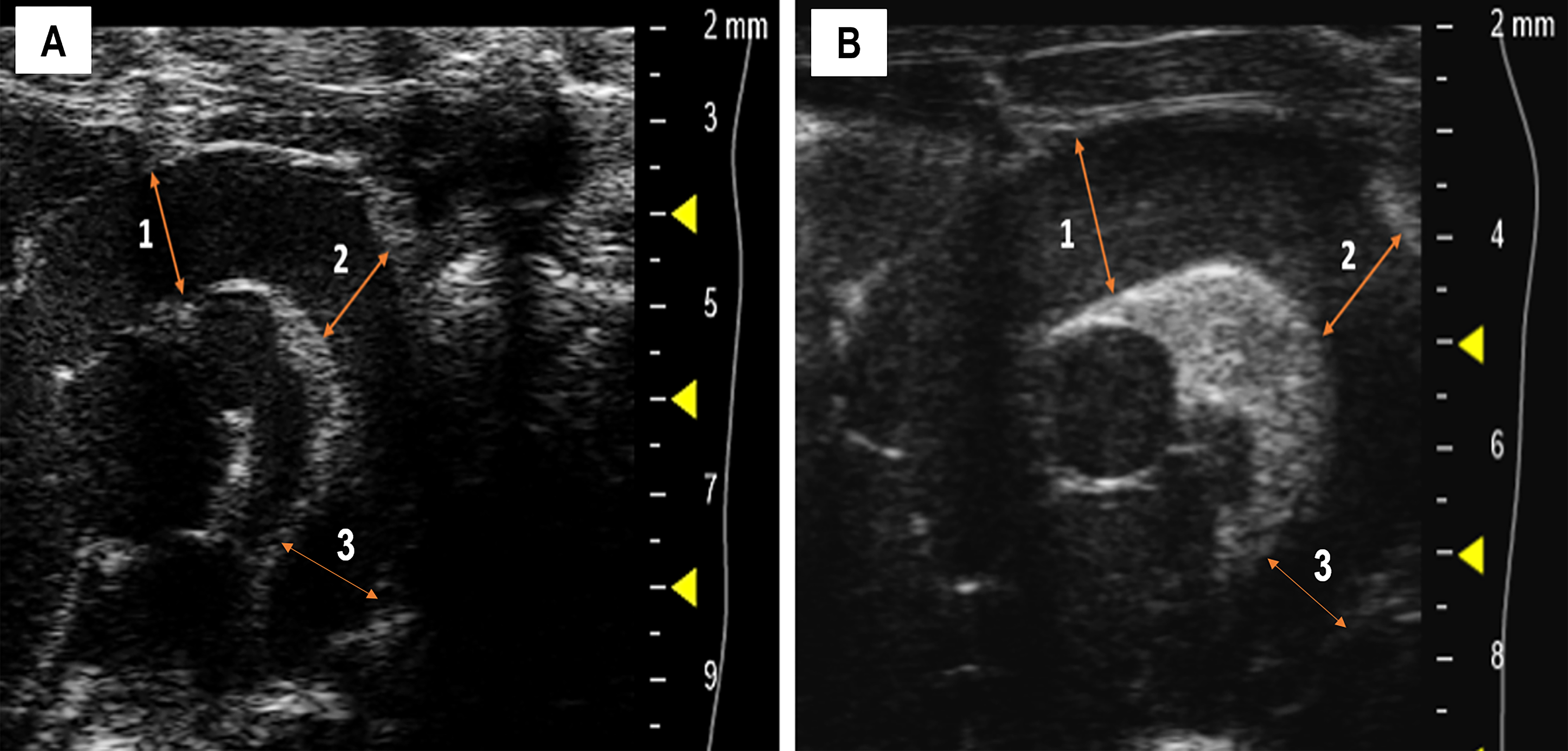

Supplement: Supplementary Figure 1 — The bi-dimensional images of (A) wild-type and (B) Marfan syndrome mice indicating the ascending aorta (1), aortic arch (2), and descending aorta (3) diameters were assessed by B-mode and measured from the suprasternal window in the longitudinal plane. [file Image_1.TIF]
