# Supplementary material for: Treatment With Methotrexate Associated With Lipid Core Nanoparticles Prevents Aortic Dilation in a Murine Model of Marfan Syndrome
Source: Front Cardiovasc Med. 2022 Jun 10;9:893774. doi: 10.3389/fcvm.2022.893774 (PMC9226570; doi:10.3389/fcvm.2022.893774)
Supplement: Supplementary Table 1 — Western blot methods used in this study. [file Table_1.docx]

Table 1 Supplementary Material. Western blot methods used in this study.

| **Primary antibody name** | **Supplier** | **Catalog number** | **Polyacrylamide/SDS gel (%)** | **Molecular**  **Weight (KDa)** | **Primary antibodies dilution** |
| --- | --- | --- | --- | --- | --- |
| Anti-adenosine A1 receptor | Abcam | Ab82477 | 10 | 36 | 1:1,000 |
| Anti-adenosine A2a receptor | Abcam | Ab3461 | 10 | 36 | 1:1,000 |
| Anti-adenosine A2b receptor | Abcam | Ab135865 | 10 | 37 | 1:1,000 |
| Anti-adenosine A3 receptor | Abcam | Ab197650 | 10 | 36 | 1:1,000 |
| Anti-adenosine deaminase | Calbiochem | AB176 | 10 | 41 | 1:1,000 |
| Anti-adenosine kinase | Abcam | Ab38010 | 10 | 41 | 1:1,000 |
| Anti-BAX | Abcam | Ab7977 | 12 | 26 | 1:500 |
| Anti-Bcl-2 | Abcam | Ab59348 | 12 | 26 | 1:1,000 |
| Anti-β-actin | Abcam | Ab5441 | 8-15 | 42 | 1:10,000 |
| Anti-cleaved-caspase 3 | Abcam | Ab2302 | 15 | 17 | 1:1,000 |
| Anti-CD3 | Abcam | Ab5690 | 12 | 23 | 1:500 |
| Anti-CD68 | Abcam | Ab125212 | 12 | 35 | 1:1,000 |
| Anti-collagen I | Abcam | Ab90395 | 8 | 139 | 1:1,000 |
| Anti-HIF-2α | Abcam | Ab199 | 8 | 118 | 1:1,000 |
| Anti-IL-1-β | Abcam | Ab82558 | 12 | 17 | 1:500 |
| Anti-IL-6 | Abcam | Ab83339 | 12 | 22 | 1:1,000 |
| Anti-MCP-1 | Abcam | Ab25124 | 12 | 25 | 1:1,000 |
| Anti-MMP2 | Abcam | Ab37150 | 8 | 72 | 1:1,000 |
| Anti-MMP9 | Abcam | Ab38898 | 8 | 82 | 1:1,000 |
| Anti-p44/42 MAP Kinase | Cell signaling | L34F12 | 10 | 42/44 | 1:1,000 |
| Anti-phospho-p44/42 MAPK (ERK1/2) | Cell signaling | E10 | 10 | 42/44 | 1:500 |
| Anti-phosphoSMAD3 | Abcam | Ab74062 | 10 | 48 | 1:500 |
| Anti-SMAD3 | Abcam | Ab40854 | 10 | 55 | 1:1,000 |
| Anti-TGF-β | Abcam | Ab66043 | 12 | 13 | 1:1,000 |
| Anti-TNF-α | Abcam | Ab1793 | 15 | 17 | 1:1,000 |
| Anti-VEGF | Abcam | Ab46154 | 12 | 23 | 1:1,000 |

# Abbreviations: BAX, Bcl-2 associated X protein; Bcl-2, B-cell lymphoma; CD3, T lymphocyte; CD68, macrophages; HIF, hypoxia-inducible factor; IL, interleukin; MCP-1, monocyte chemoattractant protein*-*1*;* MMP, metalloproteinase; TGF, transforming growth factor; TNF, tumor necrosis factor; VEGF, vascular endothelium growth factor.
